# Supplementary material for: Characterization of Rickets Type II Model Rats to Reveal Functions of Vitamin D and Vitamin D Receptor
Source: Biomolecules. 2023 Nov 19;13(11):1666. doi: 10.3390/biom13111666 (PMC10669209; doi:10.3390/biom13111666)
Supplement: Supplementary file 1 [file biomolecules-13-01666-s001.zip › biomolecules-2710289-supplementary.pdf]

(A) *Vdr*-KO 14 wk **(B) *Vdr*-KO 30 wk**

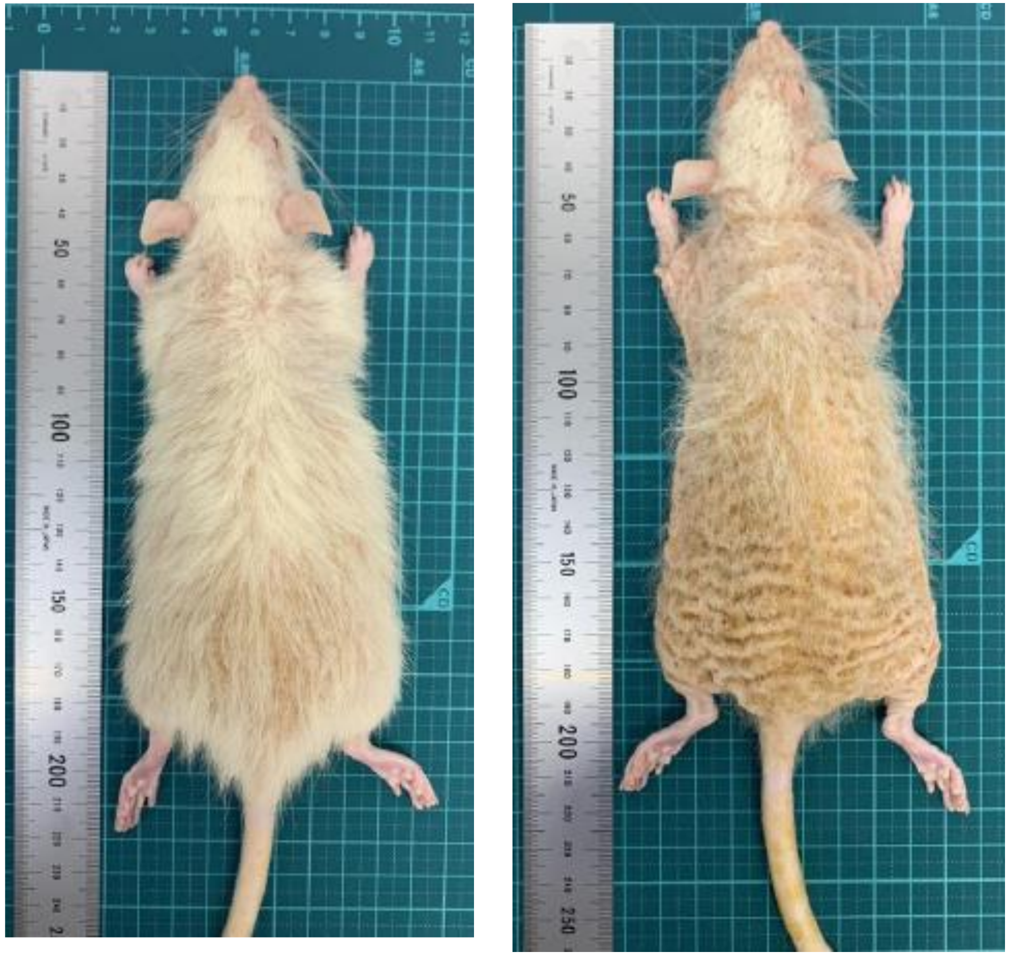

Suppl. Fig.1 Progress of alopecia and skin aging in Vdr KO rats.
Comparison between 14 weeks (A) and 30 weeks (B) after birth.

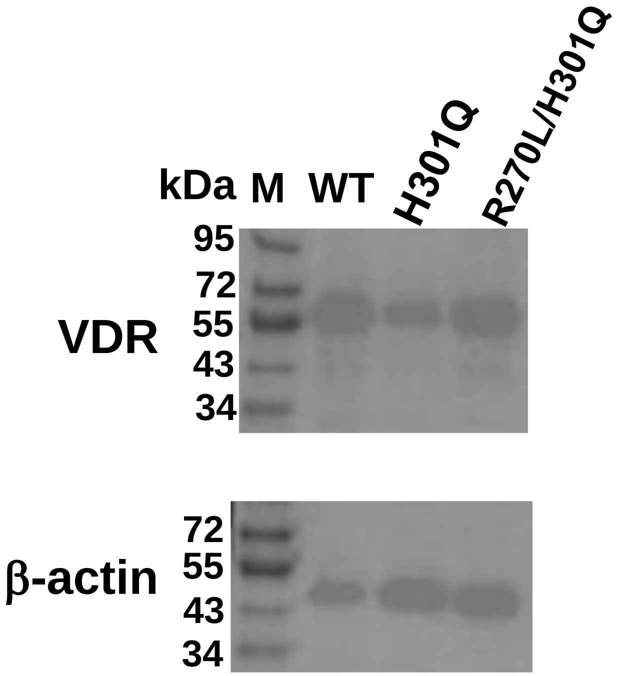

Suppl. Fig. 2
Western blot analysis of VDRs expressed in dorsal skin of WT, Vdr(H301Q), and Vdr(R270L/H301Q) rats.
